# Autocrine insulin-like growth factor 2 signaling as a potential target in the associated development of pulmonary emphysema and cancer in smokers

**DOI:** 10.1186/s41232-024-00344-3

**Published:** 2024-06-21

**Authors:** Hye-Jin Boo, Hye-Young Min, Heung-Bin Lim, Euni Lee, Ho-Young Lee

**Affiliations:** 1 Creative Research Initiative Center for Concurrent Control of Emphysema and Lung Cancer, College of Pharmacy, Seoul National University, Seoul, 08826 Republic of Korea; 2 Department of Histology, College of Medicine, Jeju National University, Jeju, 63243 Republic of Korea; 3 Natural Products Research Institute, College of Pharmacy, Seoul National University, Seoul, 08826 Republic of Korea; 4 Department of Industrial Plant Science and Technology, Chungbuk National University, Cheongju, 28644 Republic of Korea; 5 College of Pharmacy and Research Institute of Pharmaceutical Sciences, Seoul National University, Seoul, 08826 Republic of Korea

**Keywords:** Emphysema, Lung cancer, Cigarette smoking, Insulin-like growth factor 2, Calcium channel blocker

## Abstract

**Background:**

Tobacco smoking causes pulmonary inflammation, resulting in emphysema, an independent risk factor for lung cancer. Induction of insulin-like growth factor 2 (IGF2) in response to lung injury by tobacco carcinogens, 4-(methylnitrosamino)-1-(3-pyridyl)-1-butanol and polycyclic aromatic hydrocarbon benzo[a]pyrene in combination (NB), is critical for the proliferation of alveolar type 2 cells (AT2s) for lung repair. However, persistent IGF2 overexpression during NB-induced severe injury results in hyperproliferation of AT2s without coordinated AT2-to-AT1 differentiation, disrupting alveolar repair, which leads to the concurrent development of emphysema and lung cancer. The current study aims to verify the role of IGF2 signaling in the associated development of emphysema and cancer and develop effective pharmaceuticals for the diseases using animal models that recapitulate the characteristics of these chronic diseases.

**Methods:**

The pathogenesis of pulmonary emphysema and cancer was analyzed by lung function testing, histological evaluation, in situ zymography, dihydroethidium staining, and immunofluorescence and immunohistochemistry analyses utilizing mouse models of emphysema and cancer established by moderate exposure to NB for up to seven months.

**Results:**

Moderate NB exposure induced IGF2 expression in AT2s during the development of pulmonary emphysema and lung cancer in mice. Using AT2-specific insulin receptor knockout mice, we verified the causative role of sustained IGF2 signaling activation in AT2s in emphysema development. IGF2-targeting strategies, including voltage-dependent calcium channel blocker (CCB) and a neutralizing antibody, significantly suppressed the NB-induced development of emphysema and lung cancer. A publicly available database revealed an inverse correlation between the use of calcium channel blockers and a COPD diagnosis.

**Conclusions:**

Our work confirms sustained IGF2 signaling activation in AT2s couples impaired lung repair to the concurrent development of emphysema and cancer in mice. Additionally, CCB and IGF2-specific neutralizing antibodies are effective pharmaceuticals for the two diseases.

**Supplementary Information:**

The online version contains supplementary material available at 10.1186/s41232-024-00344-3.

## Background

Chronic obstructive pulmonary disease (COPD) and lung cancer are the leading causes of death worldwide, and few therapies are available [1, 2]. Emphysema, a major pathological component of COPD, is an independent risk factor for lung cancer [3–5], particularly squamous cell carcinoma [6]. Tobacco smoking (TS) is a common risk factor for COPD and lung cancer [7]. A number of molecular mechanisms induced by TS have been implicated in emphysematous lung destruction [8, 9]. Stem cell (SC) populations in the pulmonary epithelium function defend against lung injury [10], and defects or alterations in SC function may lead to the development of various pulmonary diseases [10]. The mature lung alveolar epithelium is composed of squamous type 1 cells (AT1 cells, AT1s), the main alveolar cells for gas exchange, and cuboidal type 2 cells (AT2 cells, AT2s), which prevent alveolar collapse by producing surfactants [10]. A growing body of evidence supports the SC function of AT2s in replenishing damaged alveolar epithelium [10–12]. Turnover of AT2s is low at a steady state but increases after injury to restore the damaged alveolar barrier [13, 14]. Hence, tight control of cell self-renewal and differentiation of AT2s is pivotal for maintaining lung homeostasis after TS-induced pulmonary injury.

The insulin-like growth factor (IGF) axis, which comprises two ligands (IGF1 and IGF2), three receptors (IGF1 receptor [IGF-1R], IGF2 receptor [IGF-2R], and insulin receptor [IR]), and seven IGF-binding proteins (IGFBPs, from IGFBP1 to IGFBP7) [15], plays multiple roles in the regulation of cellular growth, proliferation, and differentiation [15]. The IGF axis cross-talks with various signaling pathways involved in SCs, such as the Wnt pathway [16], which plays an essential role in SC self-renewal [17]. Levels of IGF2 expression and the ratio of IR isoforms (IR-A:IR-B) have been implicated in the regulation of a variety of SCs [18]. We have shown that exposure to two representative tobacco carcinogens (TCs), 4-(methylnitrosamino)-1-(3-pyridyl)-1-butanol (NNK) and polycyclic aromatic hydrocarbon benzo[a]pyrene (BaP), in combination (NB) through intratracheal instillation induces pulmonary inflammation, causing architectural damages in the lung [19]. As a defense mechanism to repair lung architecture against NB-induced injury, AT2s undergo active proliferation via transcriptional upregulation of IGF2 and subsequent activation of the IGF-1R/IR signaling pathway [19]. However, severe lung injury caused by daily exposure to NB for a month results in excessive IGF-1R/IR signaling activation via persistent transcriptional upregulation of IGF2 under epigenetic modulation. Consequent hyperproliferation of AT2s without coordinated AT2-to-AT1 differentiation results in impairment of the normal repair of the lung and progressive development of emphysema and lung cancer [19]. However, COPD is a progressive disease frequently associated with low-grade systemic chronic inflammation [20, 21] and airflow obstruction [22], and our findings from the NB-induced severe injury may not recapitulate the features of pathogenesis observed in the human counterpart.

This study aims to verify the role of autocrine IGF2 signaling in the TS-induced associated development of emphysema and cancer and to develop effective pharmaceuticals for the chronic pulmonary illness utilizing mouse models that can mimic aspects of biological processes during the development of the deadly diseases.

## Materials and methods

### Reagents

NNK was purchased from Toronto Research Chemicals, Inc. (North York, Ontario, Canada). Chemicals utilized in this study were procured from Sigma-Aldrich, headquartered in St. Louis, MO, USA, unless otherwise specified. Tobacco smoking extract (TSE) was prepared as described previously [23].

### Mouse experiment

The protocols for performing mouse experiments were approved by the Seoul National University Institutional Animal Care and Use Committee. The mice were allowed to consume food and water without restriction. They were also kept in a facility that was equipped with a semi-specific pathogen-free (SPF) barrier, a light-dark cycle of a twelve-hour interval, and a controlled temperature of 22±2°C. Experiments were conducted using male and female FVB/N mice aged between 4 and 12 weeks.

To investigate the effects of NB on lung injury and development of emphysema and cancer, FVB/N mice were given NNK and BaP (NB, 3 μmol each in 0.1 mL of cottonseed oil) by oral gavage twice per week for up to seven months. In addition, to examine the effect of TSE on emphysema development, FVB mice were treated with 50 μL of TSE (10 mg/mL) by oral gavage twice per week for one year and then euthanized. Conditional *Insr* knockout (*Insr* ^fl/fl^) mice on a C57BL/6J background were purchased from Jackson Laboratories (Bar Harbor, ME, USA). The *Sftpc*-CreER^T2^ mouse on a C57BL/6J background was kindly provided by Dr. Brigid Hogan (Duke University, Durham, NC, USA). These mice were backcrossed onto the FVB/N background with FVB/N mice (purchased from Japan SLC, Inc., Hamamatsu-shi, Japan) for over eight generations. For inducing Cre expression in Sftpc-positive cell populations, mice were intraperitoneally administered 0.25 mg/g body weight of tamoxifen (TM, dissolved in cottonseed oil at a concentration of 25 mg/mL) once daily for four days. Injected mice were allowed to recover for at least seven days after the last injection.

To evaluate the effects of IGF2 depletion on the initiation and development of pulmonary emphysema and lung tumorigenesis, mice were administered antibodies. Briefly, mice were given NNK and BaP (NB, 3 μmol each in 0.1 mL of cottonseed oil) by oral gavage twice per week for 12 weeks. After 12 weeks, IGF2-neutralizing antibody (R&D Systems, Minneapolis, MN, USA; 10 μg per mouse) or IgG control (10 μg per mouse) was given with or without NB by intratracheal instillation once a week for 8 weeks. To assess the impact of a calcium channel blocker on NB-induced development of pulmonary emphysema and lung cancer, mice were orally treated with vehicle or NB twice a week for 12 weeks, and, one week after NB treatment, amlodipine (0.8 mg/kg, six times a week via oral gavage) was treated in combination with NB.

### Measurement of lung function

Each mouse was weighed before anesthetizing with ketamine and xylazine. Then, a tracheostomy was conducted with an 18-gauge cannula. The cannula was connected to a computer-controlled small animal ventilator (flexiVent; Scireq, Montreal, PQ, Canada) and regular quasi-sinusoidal ventilation started at a frequency of 150 breaths/min and a tidal volume of 10 mL/kg. Before measuring lung function, deep inflation was performed by applying a pressure of 30 cmH_2_O. This step was useful for normalizing volumes in the lung. The mechanical properties of the lungs, including compliance and tissue elastance, were measured using the SanpShot-150 and Quick Prime-3 perturbations. Three readings per mouse were taken.

### Histological analysis

Paraffin sections (4 μm) and cryosections (8 μm) were used for histology. Sections were stained with hematoxylin and eosin for histological analysis. The mean linear intercept (chord)-length (MLI) method [24] was used to quantify airspace enlargement. Briefly, randomly selected fields under a magnification of 10× were evaluated from each lung. Ten lines were randomly placed on the fields, and the number of intercepts crossing the alveolar wall was counted using ImageJ software (National Institute of Health, Bethesda, MD, USA).

### Analysis of lung tumorigenesis by administration of NB

Tumor formation was evaluated and compared with that of the control group. Hematoxylin and eosin (H&E) staining was performed to measure the mean tumor number (N) and volume (V) in a blinded fashion. The tumor volume was determined using the following formula: V (mm^3^) = (long diameter × short diameter^2^)/2. The tumor burden was calculated using the following formula: mean tumor number (N) × mean tumor volume (V). The number and size of tumors were evaluated in six sections uniformly distributed throughout each lung.

### Immunofluorescence staining

Paraffin sections (4 μm) and cryosections (8 μm) were used for immunofluorescent analysis. In the case of the paraffin sections, the sections were deparaffinized and dehydrated. We then performed antigen retrieval using a citrate-based antigen unmasking solution. In the case of cryosections, the sections were fixed in 4% paraformaldehyde for 30 min at room temperature. Permeabilization was performed using 0.2% Triton X-100 in PBS for 15 min at room temperature. Blocking was performed with 10% normal serum in TBST (0.1% Tween-20 in Tris-buffered saline) for 1 h at room temperature. Primary antibodies were incubated overnight at 4°C with the following antibodies: Pdpn (1:500) (hamster, Developmental Studies Hybridoma Bank, Iowa City, IA, USA), SPC (1:200) (rabbit, Millipore, Billerica, MA, USA), MPO (1:100) (rabbit, Abcam, Cambridge, UK), F4/80 (1:100) (rat, Bio-Rad Laboratories, Hercules, CA, USA), CD4 (1:200) (rabbit, Abcam), CD8 (1:200) (rat, Abcam), Muc1 (1:500) (hamster, Thermo Fisher Scientific, Waltham, MA, USA), IGF2 (1:100) (goat, Santa Cruz Biotechnology, Dallas, TX, USA), IGF2 (1:100) (goat, R&D Systems), pIGF-1R/IR (Y1135/36, rabbit, Cell Signaling Technology, Danvers, MA, USA), and Ki67 (1:200) (rabbit, Abcam). Alexa Fluor-conjugated secondary antibodies (1:500) (Thermo Fisher Scientific or Abcam) were incubated for 1 h at room temperature. The nuclei were stained with 4,6-diamidino-2-phenylindole (DAPI, Sigma-Aldrich). Fluorescence images were acquired using Leica TCS SP8 (Leica Microsystems, Wetzlar, Germany) or Zeiss LSM700 (Carl Zeiss SMT GmbH, Oberkochen, Germany) laser scanning confocal microscopes. For co-immunofluorescence analyses of AT2-specific expression of IGF2, Ki67, and pIGF-1R/IR, we selected either SPC-specific or Muc1-specific antibodies in each experiment, considering the availability of antibodies based on their application for immunofluorescence staining and the cross-reactivity of the secondary antibodies to primary antibodies from different species.

### Immunohistochemistry

Immunohistochemistry to evaluate the level of IGF1, IGF2, IR, and IGF-1R expression in the lungs of PBS or TSE-treated mice was performed as described previously [19] with the following antibodies: IGF1 (1:100) (rabbit, Santa Cruz Biotechnology), IGF2 (1:200) (rabbit, Abcam), insulin receptor (1:100) (rabbit, Santa Cruz Biotechnology), and IGF-1R (1:100) (rabbit, Cell Signaling Technology).

### Terminal deoxynucleotidyl transferase dUTP nick end labeling (TUNEL) assay

Apoptotic DNA fragmentation was analyzed using a TUNEL assay kit (Millipore, MA, USA) according to the manufacturer’s instructions. Briefly, cryosections (8 μm) were fixed in 1% paraformaldehyde for 10 min at room temperature. After washing with PBS, the sections were post-fixed in precooled ethanol:acetic acid 2:1 for 5 min at ‒20°C. The sections were then incubated with the working strength TdT enzyme for 1 h at 37°C. Then, the sections were incubated with stop/wash buffer for 10 min at room temperature. Finally, an anti-digoxigenin conjugate was applied to the sections for 30 min at room temperature. All slides were counterstained with DAPI.

### In situ zymography for measuring matrix metalloproteinase (MMP) activity

Unfixed cryosections of the lungs (8 μm) were investigated. The fluorescein-conjugated DQ-gelatin (Thermo Fisher Scientific) diluted in low-melting agarose was placed on top of the dried sections and covered with a coverslip. Incubation was performed for 3 h at room temperature. Fluorescein isothiocyanate (FITC) fluorescence was detected with excitation at 460-500 nm and emission at 512-542 nm.

### Dihydroethidium (DHE) staining for measuring in vivo reactive oxygen species (ROS) levels

Unfixed cryosections (8 μm) were investigated. Sections were incubated with ROS-sensitive dye DHE (Thermo Fisher Scientific) for 30 min at 37°C. The slides were counterstained with DAPI. Images were acquired using a Zeiss LSM700 laser scanning confocal microscope.

### Mouse lung dissociation and flow cytometry for isolation of alveolar epithelial type 1 and 2 cells

The mice were euthanized by isoflurane inhalation. The lungs were perfused with cold PBS through the right ventricle. Lungs were inflated with 2 mL dispase (Corning Inc., Corning, NY, USA) and 1 mL of 1% low-melting agarose into trachea, then minced into small pieces in a 50-mL conical tube, and 3 mL PBS, 60 μL of collagenase/dispase, and 7.5 μL of 1% DNase I followed by shaking incubation for 45 min at 37°C. Next, the cells were filtered sequentially using 70- and 40-μm strainers and centrifuged at 1,500 rpm for 5 min at 4°C. The cell pellet was suspended in 1 mL of red blood cell (RBC) lysis solution and lysed for 1.5 min at room temperature. Then, 6 mL of basic DMEM/F12 media and 500 μL of FBS were added to the bottom of tube. After centrifugation, the cell pellet was suspended in PBS with 10% FBS for staining against alveolar epithelial type 1 and 2 cells for flow cytometry. To isolate alveolar epithelial type 1 and 2 cells, we used the following antibodies: CD45-APC (BD Biosciences, San Jose, CA, USA), CD31-APC (BD Biosciences), Sca1-APC/Cy7 (BD Biosciences), EpCAM-PE/Cy7 (BioLegend, San Diego, CA, USA), and Pdpn-PE (BioLegend). Cell sorting was performed on a FACSAria II (BD Bioscience) installed at the National Center for Inter-university Research Facilities (NCIRF) at Seoul National University.

### Real-time PCR

Real-time PCR analyses were performed as previously described [19, 25]. The relative mRNA expression was quantified using the comparative cycle threshold (CT) method, as previously described [26].

### Statistical analysis

Data are reported as mean ± SD. The values in the graphs reflect the results of multiple replicates in a representative experiment. Statistical significance was established using a two-tailed Student's t-test or one-way analysis of variance (ANOVA) in GraphPad Prism (version 10, GraphPad Software, Boston, MA, USA). There were no statistical procedures utilized to calculate sample size for animal studies. To check that the variances of two or more than three experimental groups were equal, an F-test and the Brown-Forsythe test were used, respectively. The Shapiro-Wilk test was used to see whether the data had a normal distribution. *P*-values < 0.05 were deemed statistically significant.

## Results

### Chronic exposure to NB results in the development of pulmonary emphysema and lung cancer in mice

**Fig. 1:**
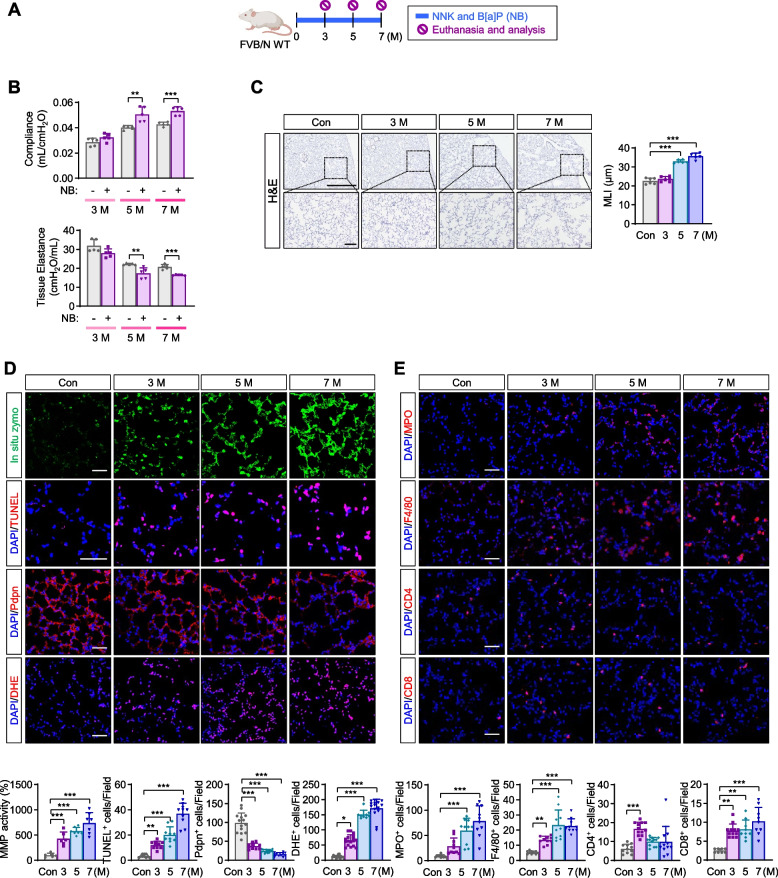
The associated development of emphysema and lung cancer by chronic NB exposure. **A** Schematic diagram of the experimental procedure (*n* ≥ 7/group). **B** Time-dependent modulation of lung function in NB-treated mice (*n* = 5/group). **C** Representative H&E images and quantitative analysis of airspace enlargement [mean linear intercept (MLI)] in the lungs of NB-treated mice (*n* = 6/group). **D**, **E** Representative immunofluorescence images and quantitative data of MMP activity, apoptosis (TUNEL^+^ cells), podoplanin (Pdpn)^+^ type I epithelial cells (AT1s), recruitment of inflammatory cells (MPO^+^ and F4/80^+^ cells), and ROS generation (DHE^+^ cells) in the lungs of NB-exposed mice (*n* = 6‒17/group). Data are the mean ± SD. *p < 0.05; **p < 0.01; ***p < 0.001 [two-tailed Student’s *t*-test (**B**); one-way ANOVA with Dunnett’s post-hoc test (**D**, **E**), Kruskal-Wallis test with Dunn’s post-hoc test (**D**, **E**)]. Scale bars: 50 μm (**C-E**)

To investigate whether IGF1R/IR signaling is implicated in the associated development of pulmonary emphysemas and cancer, we analyzed FVB/N mice that were exposed to NB in accordance with the well-established protocol for lung cancer development (3 μmol each, twice weekly via oral gavage) [27] (Fig. 1A). Up to seven months of NB exposure, mice showed time-dependent increases in emphysematous phenotypes in the lungs, including diminution in lung function (i.e., increase in compliance and decrease in tissue elastance) (Fig. 1B). Additionally, they also displayed indicators of destruction of lung parenchyma, including enlarged alveolar airspace quantified by mean linear intercept (MLI) (Fig. 1C), increased MMP activity measured by in situ zymography using fluorescein-conjugated DQ gelatin, pulmonary cell apoptosis measured by TUNEL assay, and decreased Pdpn^+^ AT1s measured by immunofluorescence staining (Fig. 1D). It has been believed that emphysema is driven by oxidative stress and chronic inflammation [28]. Indeed, time-dependent increases in dihydroethidium (DHE)^+^ ROS-producing cells, myeloperoxidase (MPO)^+^ polymorphonuclear neutrophils (PMNs), F4/80^+^ macrophages, CD4^+^ T cells, and CD8^+^ T cells were observed in the lungs of NB-treated mice (Fig. 1E). Tumor nodules were detected in 100% of mice (*n* = 11) exposed to NB for 7 months, and time-dependent increases in the number of tumors, tumor volume, and tumor burden were observed (see Supplementary Fig. 1, Additional file 1). These results suggest that chronic inflammation caused by sustained exposure to NB results in the associated development of pulmonary emphysema and cancer.

### Upregulation of IGF2 in AT2s is associated with NB-induced pulmonary emphysema development

**Fig. 2:**
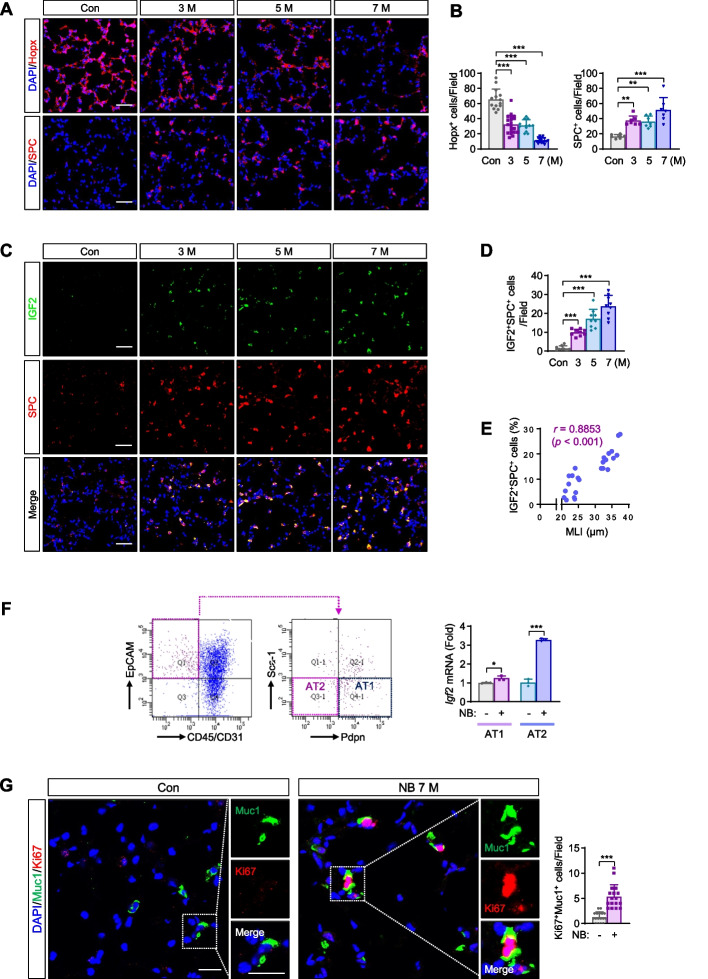
The impact of NB treatment on the level of alveolar epithelial cells, IGF2 expression of type II alveolar epithelial cells, and their proliferation. **A**, **B** Representative immunofluorescence (IF) images and quantitative analysis of inverse modulation between Hopx^+^ type I alveolar epithelial cells (AT1s) and SPC^+^ type II alveolar epithelial cells (AT2s) after NB exposure (*n* = 7‒20/group). **C**, **D** Representative IF images and quantitative analysis of upregulation of IGF2 in SPC^+^ AT2s (*n* = 9/group). **E** Spearman correlation between the level of IGF2 in SPC^+^ AT2s and the severity of airspace enlargement (*n* = 24/group). **F** *Left.* Gating strategy for isolating murine AT1s and AT2s. *Right.* Real-time PCR analysis showing elevated *Igf2* expression in NB-treated AT2s (*n* = 3/group). **F** Representative IF images and quantitative analysis showing upregulation of the proliferation of AT2s (Ki67^+^Muc1^+^ cells) in the lungs after treatment with NB for seven months (*n* = 17/group). Data are the mean ± SD. *p < 0.05; **p < 0.01; ***p < 0.001 [one-way ANOVA with Dunnett’s post-hoc test (**B**, **D**); two-tailed Student’s *t*-test (**F**, **G**)]. Scale bars: 50 μm (**A**, **C**), 20 μm (**G**)

As COPD involves an abnormal repair mechanism against lung injury caused by environmental toxicants [3], we analyzed the effects of NB exposure on the level of AT2s (the SPC^+^ population) and AT1s (the Hopx1^+^ population), which are cellular components that function as alveolar stem/progenitor cells for epithelial cell regeneration [10]. Immunofluorescence (IF) staining of lung sections (Fig. 2A) and associated statistical analysis (Fig. 2B) revealed time-dependent increases in AT2s and concomitant decreases in AT1s as early as three months after NB exposure. Next, we assessed whether IGF2 expression was implicated in emphysema development in NB-exposed mice. We found a time-dependent increase in the number of pulmonary AT2s expressing IGF2 after NB exposure (Fig. 2C, D). In contrast, IGF2 expression in AT1s remained unchanged even after seven months of exposure to NB (see Supplementary Fig. 2, Additional file 2). Notably, the severity of emphysema assessed by MLIs was significantly correlated with the degree of AT2s expressing IGF2 (Fig. 2E). Real-time PCR analysis of fluorescence-activated cell sorting (FACS)-purified AT1s and AT2s further revealed the transcriptional upregulation of IGF2 in AT2s rather than in AT1s in NB-exposed mice (Fig. 2F). To determine whether the increased number of AT2s was due to enhanced proliferation, we analyzed the level of Ki67 positivity (an indicator of cell proliferation [29]) in AT2s. Approximately 20% of AT2s were Ki67^+^ after seven months of NB treatment (Fig. 2G), indicating that AT2 expansion after long-term NB exposure resulted from proliferation.

### IGF2 is upregulated in AT2s during emphysema development caused by prolonged exposure to TS extracts

**Fig. 3:**
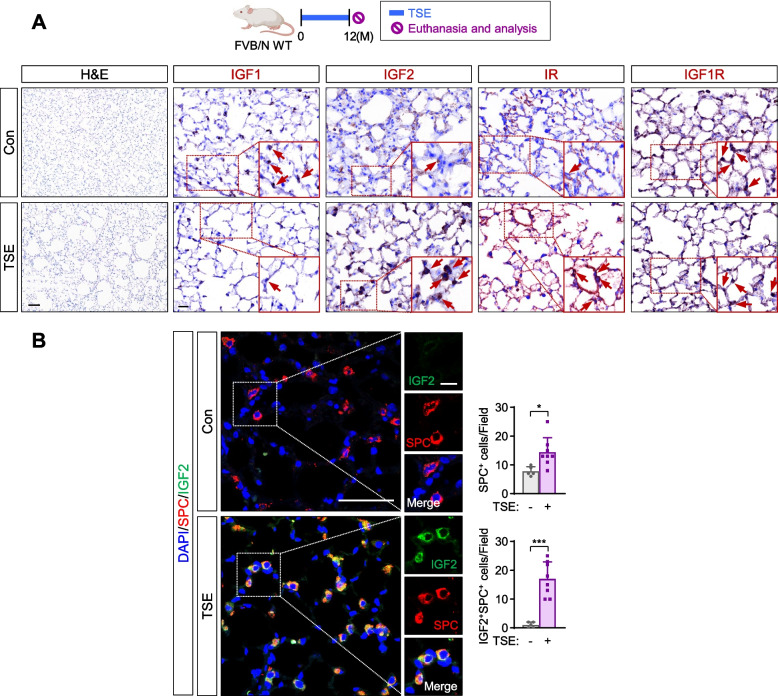
Induction of IGF2 expression in AT2s by treatment with TSE. **A** Representative H&E or immunohistochemistry (IHC) images showing airspace enlargement and changes in the expression of IGF1, IGF2, IR, and IGF-1R after treatment with TSE for 12 months. **B** Representative immunofluorescence images and quantitative analyses showing increases in the IGF2 expression in SPC^+^ AT2s (IGF2^*+*^SPC^*+*^ cells) by exposure to TSE for 12 months (*n* = 5 or 8/group). Data are the mean ± SD. ***p < 0.001 (two-tailed Student’s *t*-test). Scale bars: 25 μm (**A**, H&E images); 100 μm (**A**, IHC images); 50 μm (**B**); 10 μm (**B**, insets)

Animal models of emphysema induced by exposure to TS or TSE would more precisely represent the disease shown in humans than those in which emphysema is induced by NB. Hence, we analyzed the expression levels of IGF axis components in lung tissues of mice wherein emphysematous diseases was induced by exposure to TSE for one year. Consistent with the observation in mice in which severe lung injury caused by chronic exposure to NB, mice with TSE-induced emphysematous changes in the lung showed upregulated IGF2 expression (Fig. 3A). Robustly increased numbers of AT2s, especially IGF2^+^ AT2s, were also observed in mice with TSE-induced emphysematous lung lesions (Fig. 3B). These results suggest that prolonged NB exposure induces sustained overexpression of IGF2 and activation of IGF-1R/IR pathway, which in turn causes the development of pulmonary emphysema. We found that lung tumor development was rare in the TSE-exposed mice, probably due to insufficient doses of essential TS components, including NNK and BaP.

### Autonomous IGF2 signaling activation in AT2s leads to the development of emphysema

**Fig. 4:**
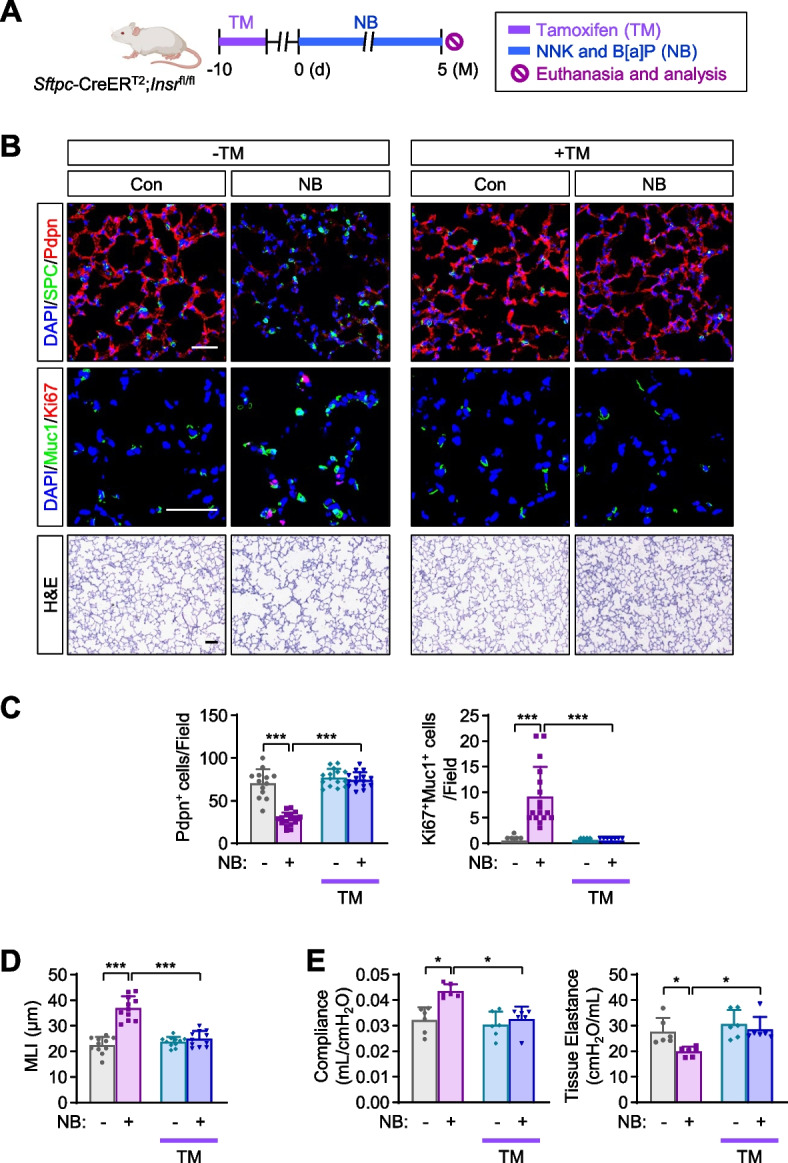
Attenuation of NB-induced emphysematous features by *Insr* knockout in type II alveolar epithelial cells. **A** Schematic diagram of the experimental procedure. **B** Representative H&E and immunofluorescence (IF) images of changes in the level of Pdpn^+^ AT1s and SPC^+^ AT2s, the proliferation of AT2s (Ki67^+^ in Muc1^+^ AT2s), and airspace enlargement. **C** Quantitative analyses of changes in the level of Pdpn^+^ AT1s and the proliferation of AT2s (Ki67^+^ in Muc1^+^ AT2s) (*n* = 11‒17/group). **D** Quantitative analysis of airspace enlargement (*n* = 11/group). **E** Changes in lung function (*n* = 6/group). Data are the mean ± SD. *p < 0.05; **p < 0.01; ***p < 0.001 (one-way ANOVA with Dunnett’s post-hoc test). Scale bars: 25 μm (**B**, H&E images); 50 μm (**B**, IF images)

We investigated whether activation of IGF2 signaling in AT2s is responsible for NB-induced pathogenesis. To this end, we assessed whether deletion of the IGF2 receptor in AT2s would attenuate NB-mediated proliferation of AT2s alongside emphysematous phenotypes using *Sftpc*-CreER^T2^;*Insr* ^fl/fl^ mice [30]. TM-induced *Insr* deletion in the AT2s of *Sftpc*-CreER^T2^;*Insr* ^fl/fl^ mice prior to NB exposure (Fig. 4A) significantly decreased the number of Ki67^+^ AT2s and restored the AT1 population in the lungs of mice exposed to NB for 5 months (Fig. 4B, C). Moreover, NB-induced emphysematous features, including enlarged alveolar airspace (i.e., mean chord length [24]) (Fig. 4B, D) and diminution of lung function (i.e., increase in compliance and decrease in tissue elastance) (Fig. 4E), were also significantly attenuated in mice with TM-mediated IR deletion. These findings suggest that autonomous IGF2-IR signaling activation in NB-exposed AT2s leads to the development of emphysema.

### Blockade of IGF2 signaling activation by neutralizing monoclonal antibody impairs NB-induced concurrent development of emphysema and lung cancer

**Fig. 5:**
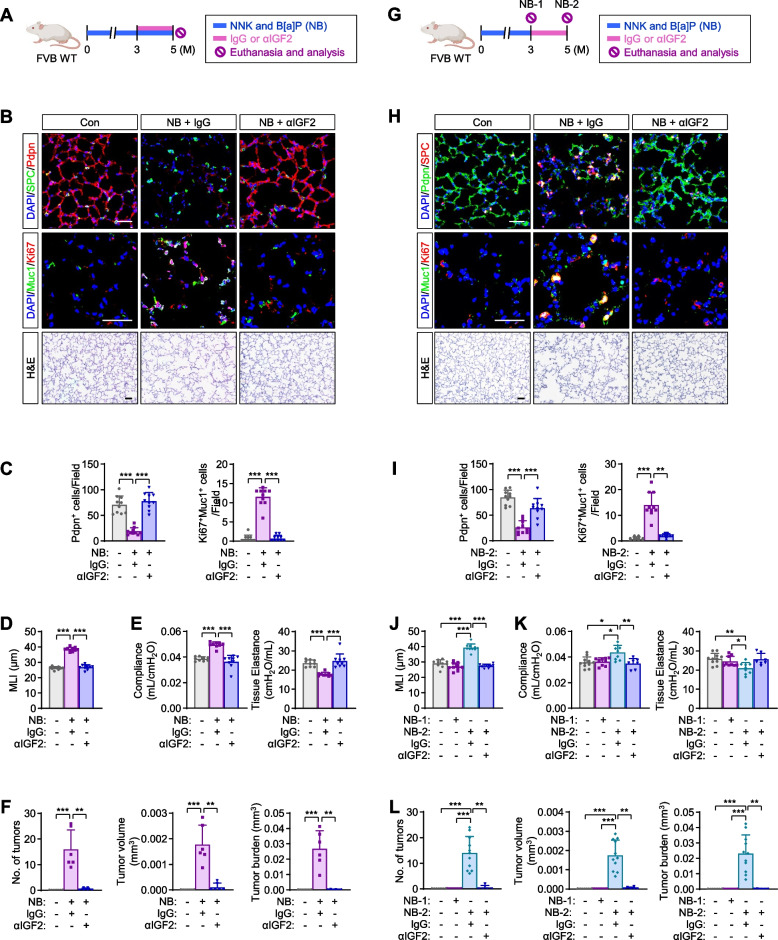
Blockade of NB-induced development of emphysema and lung cancer by targeting IGF2. **A** Schematic diagram of the experimental procedure of intratracheal treatment with IGF2 mAb in combination with NB after three-month NB treatment (the current smoker model). **B** Representative H&E and immunofluorescence (IF) images of changes in the level of Pdpn^+^ AT1s and SPC^+^ AT2s, the proliferation of AT2s (Ki67^+^ in Muc1^+^ AT2s), and airspace enlargement. **C** Quantitative analyses of changes in the level of Pdpn^+^ AT1s and the proliferation of AT2s (Ki67^+^ in Muc1^+^ AT2s) (*n* = 10/group). **D** Quantitative analysis of airspace destruction (*n* = 10/group). **E** Changes in lung function (*n* = 9/group). **F** Quantitative analyses of tumor formation (*n* = 6 or 7/group). **G** Schematic diagram of the experimental procedure of intratracheal two-month treatment with IGF2 mAb without NB after a three-month NB treatment (the ex-smoker model). NB-1 indicates the NB group that was euthanized and analyzed after three-month NB treatment. NB-2 indicates the NB group that was euthanized and analyzed at the same time as the IGF2 mAb treatment group (two- months without NB after three months of NB treatment). **H** Representative H&E and IF images of changes in the level of Pdpn^+^ AT1s and SPC^+^ AT2s, the proliferation of AT2s (Ki67^+^ in Muc1^+^ AT2s), and airspace enlargement. **I** Quantitative analyses of changes in the level of Pdpn^+^ AT1s and the proliferation of AT2s (Ki67^+^ in Muc1^+^ AT2s) (*n* = 10/group). **J** Quantitative analysis of airspace destruction (*n* = 10/group). **K** Changes in lung function (*n* = 7‒11/group). **L** Quantitative analyses of tumor formation (*n* = 6‒11/group). Data are the mean ± SD. *p < 0.05; **p < 0.01; ***p < 0.001 [one-way ANOVA with Dunnett’s post-hoc test (**C-E**, **I-K**) or Kruskal-Wallis test with Dunn’s post-hoc test (**C**, **F**, **I**, **L**)]. Scale bars: 25 μm (H&E images); 50 μm (IF images)

Next, we assessed the effects of IGF2 deprivation strategies on NB-induced pulmonary emphysema and tumor development. To this end, after three-month-exposure to NB, neutralizing monoclonal antibody against IGF2 (αIGF2 mAb) or control IgG (IgG) was administered in combination with NB (mimicking current smokers) (Fig. 5A). All mice were sacrificed after five months. IF analysis of lung tissues and associated statistical analysis revealed that αIGF2 mAb treatment significantly attenuated the NB-induced increases in proliferating AT2s and decreases in AT1 population in the lungs (Fig. 5B, C). Histological analysis of hematoxylin and eosin (H&E) stained lung tissues revealed that alveolar airspace quantified by MLI was markedly normalized by the administration of αIGF2 mAb (Fig. 5B, D). Mice exposed to NB for five months in the presence of αIGF2 mAb also showed significant restoration of lung function (i.e., increase in compliance and decrease in tissue elastance) (Fig. 5E) and decreases in lung tumor multiplicity, volume, and burden in the NB-exposed mice (Fig. 5F).

To corroborate the severity of lung pathology induced by chronic NB exposure, we evaluated whether NB-induced emphysema-like tissue destruction and tumor formation could progress even after NB withdrawal (mimicking ex-smokers). To this end, we assessed the impact of two months of withdrawal after three months of repetitive NB exposure (Fig. 5G). We observed that the NB-induced changes in proliferating AT2s, AT1 population (Fig. 5H, I), and MLI (Fig. 5H, J) within the distal lung tissue and lung function (Fig. 5K) observed at three months progressed even in the absence of NB for two months. Moreover, lung tumor formation, measured by multiplicity, volume, and burden, markedly increased even 2 months after NB withdrawal (Fig. 5L). Notably, the progression of the pathologic changes due to the 3 months of NB exposure were all retarded by the administration with αIGF2 mAb (Fig. 5H-L). We conducted immunofluorescence staining of the lung tissues that had NB exposure for 3 or 5 months and those from mice that had 2 months NB cessation after the NB exposure for 3 months. We found that the increased IGF2 expression in AT2s within the alveolar epithelium caused by exposure to NB for 3 months was sustained even after NB withdrawal for 2 months (see Supplementary Fig. 3, Additional file 3).These results suggest that chronic NB exposure leads to sustained IGF2 signaling activation, which contributes to the development of COPD and lung tumors. Accordingly, IGF2 signaling is a potential target for blocking the NB-induced development of pulmonary diseases in both current and ex-smokers.

### Administration of a calcium channel blocker rescues from NB-induced development of emphysema and lung cancer

**Fig. 6:**
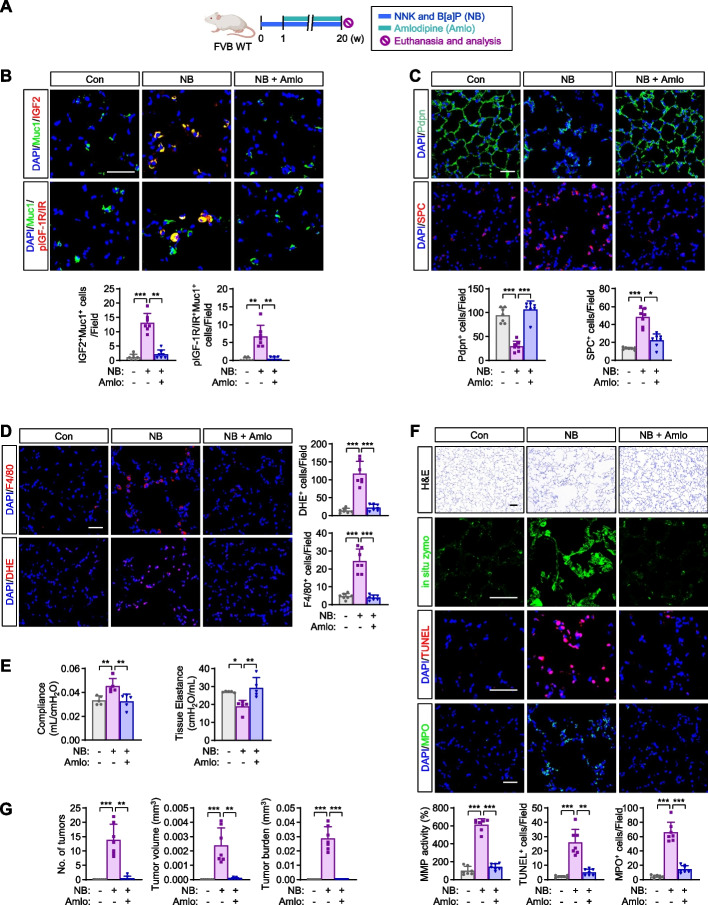
Amelioration of N/B-induced development of emphysema and lung cancer by treatment with a calcium channel blocker. **A** Schematic diagram of the experimental procedure. **B** Representative immunofluorescence (IF) images and quantitative analysis of the level of IGF2 and pIGF-1R/IR in Muc1^+^ AT2s (*n* = 7/group). **C** Representative IF images and quantitative analysis of the level of Pdpn^+^ AT1s and SPC^+^ AT2s (*n* = 7/group). **D** Representative immunofluorescence (IF) images and quantitative analyses of the recruitment of inflammatory cells (MPO^+^ and F4/80^+^ cells) (*n* = 7/group). **E** Changes in lung function (*n* = 5/group). **F** Representative H&E images of airspace destruction and representative IF images and quantitative analyses of MMP activity, apoptosis (TUNEL^+^ cells), podoplanin (Pdpn)^+^ type I epithelial cells (AT1s), and ROS generation (DHE^+^ cells) (*n* = 7/group). **G** Quantitative analyses of tumor formation (*n* = 7/group). Data are the mean ± SD. *p < 0.05; **p < 0.01; ***p < 0.001 [one-way ANOVA with Dunnett’s post-hoc test (**B-F**), Brown-Forsythe and Welch ANOVA test (**D**, **E**), or Kruskal-Wallis test with Dunn’s post-hoc test (**G**)]. Scale bars: 25 μm (**E**, H&E images); 50 μm (**B-E**, IF images)

As the blockade of voltage-dependent calcium channel (VDCC)-mediated Ca^2+^ influx suppresses NNK-induced IGF2 exocytosis and IGF-1R activation [25], we hypothesized that blockade of IGF2 secretion using clinically available calcium channel blocker (CCB), such as amlodipine (Amlo), would prevent the development of NB-induced emphysema. We then performed an animal experiment in which mice were administered NB either alone or in combination with Amlo, as illustrated in Fig. 6A. We observed that Amlo treatment significantly suppressed NB-induced IGF-1R/IR activation in AT2s in the lungs of NB-exposed mice (Fig. 6B). Additionally, the reduction in AT1s (the Pdpn^+^ population) and hyperproliferation of AT2s were nearly restored to levels similar to those observed in vehicle-treated control mice following treatment with Amlo (Fig. 6C). Amlo administration significantly attenuated NB-induced airspace enlargement (Fig. 6D) and impaired lung function (Fig. 6E). NB-induced emphysematous changes, including increases in MMP activity, pulmonary cell apoptosis, recruitment of MPO^+^ neutrophils and F4/80^+^ macrophages, and ROS production, were also significantly ameliorated by treatment with Amlo (Fig. 6F). Moreover, consistent with the results in our previous study [25], treatment with Amlo significantly suppressed NB-induced increases in lung tumor multiplicity, tumor volume, and tumor burden (Fig. 6G). These results suggest the potential utility of CCB intake for the prevention of tobacco-induced pulmonary emphysema and lung cancer.

### CCB intake and risk of COPD

To further investigate whether the regulation of intracellular Ca^2+^ suppresses TS-induced pulmonary emphysema development, we performed a quantitative assessment of the cross-sectional association between CCB prescription and emphysema diagnosis by analyzing the Korean Health Insurance Review and Assessment Service-National Patient Sample (HIRA-NPS) database collected between 2012 and 2014. During the three-year study period, the estimated average study population from the HIRA-NPS database was ~38 million per year (see Supplementary Table 1, Additional file 4). Of these, our study population was ~1.38 million per year. The mean age of the study population was 46.86 years old (SD = 16.91), and 52.25% were female. Prescriptions with dihydropyridine and non-dihydropyridine CCB were found in 8.52% and 0.80% of patients, respectively, and the prevalence of COPD-related diagnoses was 1.37% (see Supplementary Table 1, Additional file 4). When the association between COPD-related diagnoses and CCB use was evaluated, our findings showed that sex, age, insurance scheme, and the presence of CCB prescriptions were significant predictors of COPD-related diagnoses (Table 1). Male patients were more likely to be associated with COPD-related diagnosis (OR_adj_ = 2.25; 95% CI 2.20–2.29) than female patients (Table 1). As patient age increased, the likelihood of being diagnosed with COPD also increased (Table 1). Beneficiaries of Medicaid (OR_adj_ = 2.09; 95% CI 2.02–2.16) or Veterans Healthcare (OR_adj_ = 2.24; 95% CI 2.10–2.38) were more likely to be associated with COPD-related diagnosis (Table 1). Patients with a dihydropyridine-CCB prescription were less likely to have a COPD-related diagnosis (OR_adj_ = 0.86; 95% CI 0.84–0.88) (Table 1). These findings indicate an inverse association between CCB use and COPD diagnosis. In addition, dihydropyridine CCB can be the first choice for treatment of patients with both hypertension and emphysema.

**Table 1:** Predictive factors associated with COPD and emphysema diagnosis of the study population of NPS 2012-2014

**Factors**	ORadj (95% CI)*
***Gender***	
**Female**	Reference
**Male**	2.246 (2.201-2.293)
***Age (years old)***	
**19 - 34**	Reference
**35 – 49**	2.260 (2.115-2.416)
**50 – 64**	8.276 (7.791-8.791)
**65 - 79**	27.412 (25.794-29.132)
**80 and older**	46.777 (43.854-49.896)
***Public insurance scheme***	
**Health Insurance**	Reference
**Medicaid**	2.086 (2.020-2.155)
**Veteran Healthcare**	2.237 (2.101-2.382)
***Hypertension***	
**Yes**	Reference
**No**	0.786 (0.767-0.806)
***Angina Pectoris***	
**Yes**	Reference
**No**	0.465 (0.453-0.477)
***Calcium channel blockers (CCB) type***	
**No CCB**	Reference
**Dihydropyridine CCB**	0.861 (0.837-0.884)
**Non-dihydropyridine CCB**	1.128 (1.069-1.190)

^*^ *p* < 0.05

## Discussion

**Fig. 7:**
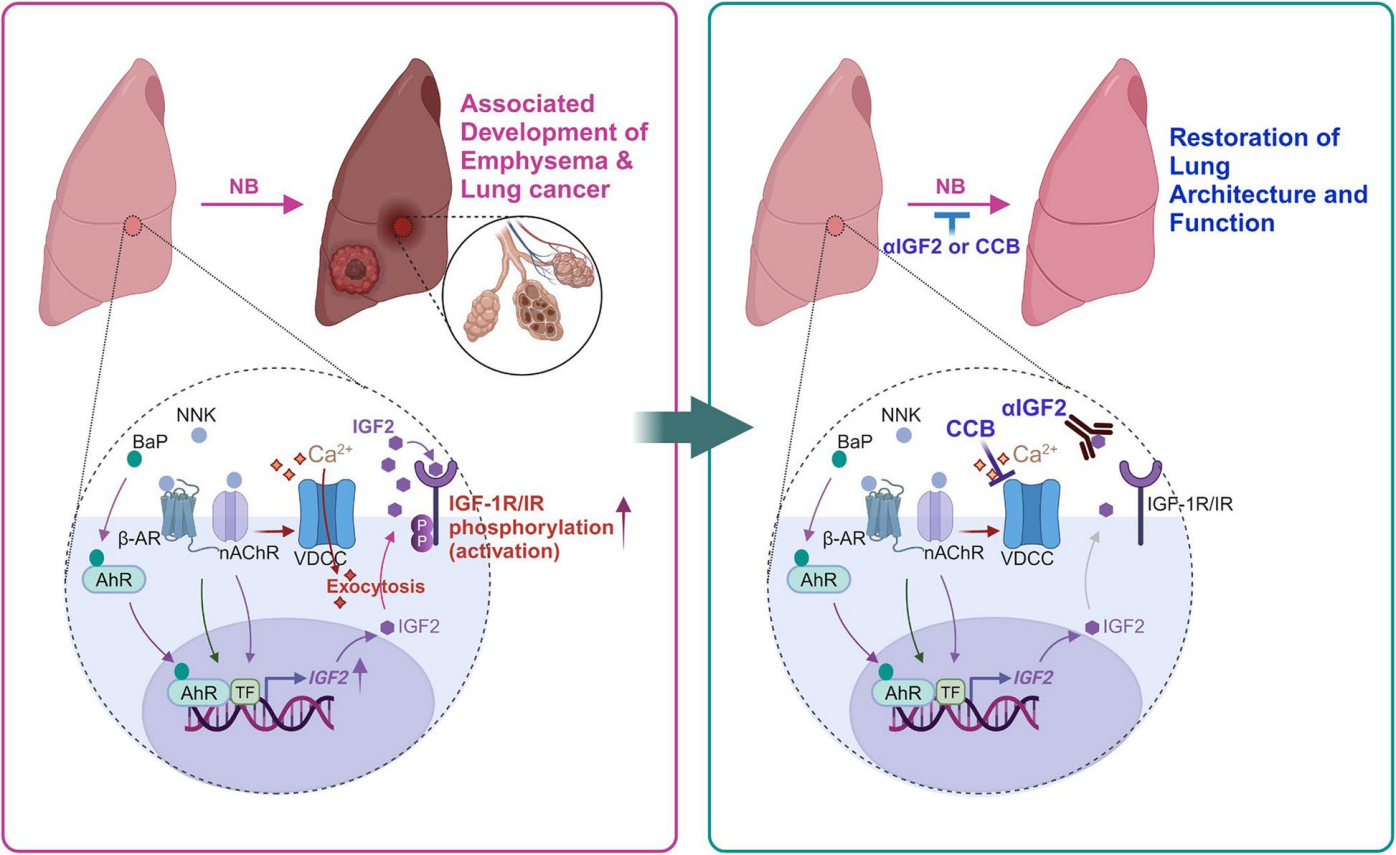
Illustration of the summary of the results. Chronic NB treatment induces persistent upregulation of IGF2 expression through NNK/β-AR, NNK/nAchR, and BaP/AhR-mediated signal transduction cascades and facilitates IGF2 exocytosis through nAChR-mediated membrane depolarization and subsequent VDCC-mediated Ca^2+^ influx. The consequent overactivation of IGF-1R/IR signaling results in the disruption of the stem cell function of AT2s, causing poor recovery of lung architecture against NB-mediated injury and the transformation of normal lung epithelial cells into malignant cells. These overall events ultimately lead to the concurrent development of emphysema and lung cancer. Abrogation of IGF2 availability by using IGF2-specific neutralizing mAbs or blocking VDCC-mediated Ca^2+^ influx by using CCB alleviates NB-mediated associated development of emphysema or lung cancer

TS has gained extensive attention as a risk factor for both COPD and lung cancer [31]. Therefore, identifying key pathophysiological mechanisms by which TS components induce the two pulmonary diseases and developing effective strategies targeting the mechanisms would be rational approaches for the treatment of the diseases. We have previously shown that severe lung injury caused by highly frequent exposure to NB (a combination of NNK and BaP) through intratracheal instillation induces sustained overexpression of IGF2 in AT2s, resulting in excessive proliferation without balanced differentiation and interfering with the appropriate recovery of the alveolar structure [19]. Epigenetic mechanisms involving the upregulation of DNA methyltransferase 3A (DNMT3A) are attributable to IGF2 overexpression after sustained NB exposure [19]. In the current study, we employed better disease models to adequately characterize the pathogenesis of the two pulmonary diseases. Our study demonstrated that long-term lung injury in response to exposure to TSE or NB for up to a year induced persistent IGF2 overexpression, which in turn, in an autocrine manner, induced hyperproliferation of AT2s, causing impairment of the normal repair of the lung and progressive development of emphysema and lung cancer (Fig. 7). Our findings clearly show that altered IGF2-IR signaling in AT2s links TS to the pathogenesis of these two pulmonary diseases.

COPD and cancer are frequent comorbidities mediated by lung inflammation primarily caused by TS [6]. Smokers with airflow obstruction are expected to develop up to five times more lung cancers than those with normal lung function [31, 32], which supports the role of premature cellular aging, telomere shortening, oxidative stress, genetic predisposition, altered expression of growth factors, and activation of intracellular pathways in both COPD and lung cancer [33]. In addition to these changes, the epigenetic regulation of gene expression through DNA methylation, histone modifications, and microRNA expression plays an essential role in the development of cancer and COPD [34, 35]. COPD involves the abnormal repair of lung injury caused by the inhalation of toxic particles and gases [3]. AT2s with SC functions can restore the alveolar structure and function after lung injury, including TS-induced injury [10]. AT2s are also a potential source for cancer development [12]. Therefore, alterations in the signaling pathways involved in the SC function of AT2s may link COPD and cancer and provide possible novel targets for the treatment of the associated development of COPD and lung cancer. We demonstrated that the induced expression of IGF2 in AT2s is essential for their SC function against NB-induced alveolar injury [19]. Despite the beneficial role of IGF2 in lung recovery against NB-induced acute injury, our current data using wild-type and conditional IR-knockout mouse models showed that prolonged NB exposure induces IGF2 overexpression, which results in the hyperproliferation of AT2s, ultimately evoking emphysematous phenotypes. Therefore, the lack of an intricate balance of signals that control the proliferation and differentiation of AT2s can result in pulmonary diseases, particularly emphysema. AT2s are generally regarded as alveolar progenitors [36], and epithelial components in smokers, particularly in AT2 clusters, are heterogeneous [36]. It has been shown that subsets of AT2s play distinct roles in maintaining lung alveolar structure and function. For example, the CD44^high^ subpopulation of AT2s regulates immune signaling pathways, and disruption of these pathways impaired the SC function of AT2s [37]. In addition, a subset of AT2s (IFN-γ-responsive CD66^+^ AT2s) displays stem-cell-like characteristics, and the level of this population was found to be decreased in COPD lungs [38]. IFN-γ was found to increase apoptosis and decrease Ki67 positivity in alveolospheres [39]. Therefore, IFN-γ-responsive SPC^+^ AT2s could be a subset that was decreased in response to IFN-γ in the pulmonary environment in patients with COPD. In contrast, IGF2 signaling is mitogenic and plays an important role in cell survival [40]. Therefore, external harmful stimuli, such as tobacco smoking, could increase IGF2^+^ AT2s due to their resistance to cell death and their role in lung regeneration and repair. In line with our findings, SPC-expressing AT2s undergo proliferation and differentiation during bacterial pneumonia along with Hippo pathway activation, which may contribute to the newly formed alveolar epithelium [41]. In this study, mice lacking Hippo signaling components in AT2s cells showed prolonged pulmonary inflammation and delayed alveolar epithelial regeneration during bacterial pneumonia. Therefore, it is likely that AT2s appear to be basically heterogeneous and exhibit various phenotypes and functions depending on the types of stimuli or surrounding circumstances. Additional studies are required to characterize the specific AT2 subpopulations at single-cell-based levels.

AT2-specific overexpression of IGF2 and IGF-1R/IR signaling activation was also observed in emphysema and tumor lesions of the lungs in patients with a history of TS [19, 42]. Therefore, our findings of NB-mediated pathobiology in a mouse model can be extrapolated to humans, particularly to the associated development of pulmonary emphysema and cancer in smokers [7, 43]. Notably, the pulmonary pathogenesis due to current or prior NB exposure progressed even after the cessation of NB exposure but was prevented in mice by an anti-IGF2 neutralizing antibody. These results suggest that the capacity for indefinite proliferation through dysregulation of IGF2 signaling predisposes individuals to emphysema and lung cancer. This concept may also explain why most patients with moderate COPD or tumors progress to severe disease even after absolute TS cessation [44]. In this setting, clinically available strategies that allow careful control of IGF2 expression within the normal range in the AT2 SC pool may be critical for preventing the pathogenesis of the two TS-induced pulmonary diseases. Based on our previous findings, including 1) NNK-induced exocytosis of IGF2 via VDCC-intervened Ca^2+^ influx [25, 42]; 2) the efficacy of antagonizing Ca^2+^ signaling in preventing NB-induced lung tumor formation in vivo [19], and 3) reduced lung cancer diagnosis via clinically available CCB medication, we hypothesized that chronic NB exposure induces excessive IGF2 signaling in AT2s through at least two independent but comparable mechanisms. Accordingly, the suppression of Ca^2+^ influx via CCB may offer new opportunities to prevent both emphysema and lung cancer. Indeed, administration of CCB effectively suppressed NB-induced emphysema development in mice. Finally, a cross-sectional evaluation of insurance claims data in Korea showed that prescription of CCB was less likely to be associated with COPD after controlling for age, sex, and insurance scheme among patients with hypertension-associated disorders or angina-related diseases. Big data, such as HIRA-NPS, may allow cross-sectional evaluations to identify associations between drug exposure and clinical outcomes. As the majority of Koreans are enrolled in this national health insurance system, the generalization of the findings from such evaluations could also be easily applied to the overall Korean population compared to other healthcare systems.

## Conclusions

We demonstrated that chronic inflammation in the lungs induced by NB exposure shunts AT2 proliferation from a resource for alveolar barrier regeneration to a common origin of emphysema and tumor development through sustained activation of IGF2 signaling. Given that 15–20% of emphysema and lung cancers worldwide are TS-related [45], NB activation of the IGF2-IGF-1R/IR signaling pathway may help us understand the pathogenesis underlying the development of these two deadly diseases in smokers. Additionally, our results highlight the use of specifically targeted therapies that control the bioavailability of IGF2 as a potential preventive strategy to reduce the risk of emphysema and cancer. Considering that CCBs, which have been used as a therapeutic strategy for the treatment of hypertension [46], are clinically available, our findings have significant clinical implications for controlling the two deadly pulmonary diseases in current smokers and ex-smokers. While the exact mechanism(s) underlying the associated development of lung cancer and COPD are currently unknown, our data shed new light on the emerging concept that diseases are closely linked to IGF2-mediated signaling in AT2s. Additionally, repositioning calcium signaling-blocking drugs such as CCBs is a novel strategy for targeting IGF2 signaling to prevent both emphysema and lung cancer without the potential deleterious effects of metabolic disorders.

### Supplementary Information

Additional file 1: Supplementary Fig. 1. Quantitative analyses of tumor formation NB-treated mice (*n* = 7/group). Data are the mean ± SD. *p < 0.05; **p < 0.01; ***p < 0.001 (Kruskal-Wallis test with Dunn’s post-hoc test).

Additional file 2: Supplementary Fig. 2. Representative IF images showing IGF2 expression in SPC^+^ AT2s and Pdpn^+^ AT1s. Scale bars: 50 μm.

Additional file 3: Supplementary Fig. 3. Representative IF images and quantitative analysis (*n* = 7) showing the regulation of IGF2 expression in mice treated with NB for 3 months, those treated with NB for 3 months and withdrawn from NB treatment for 2 months, or those treated with NB for 5 months. Data are the mean ± SD. *p < 0.05; **p < 0.01; ***p < 0.001 (one-way ANOVA with Dunnett’s post-hoc test). Scale bars: 50 μm. 10 μm (insets).

Additional file 4: Supplementary Table 1. Demographic characteristics of the study population.

## Data Availability

All data generated or analyzed during this study are included in this published article and its supplementary material files.

## Abbreviations

AT1s: alveolar type 1 cells
AT2s: alveolar type 2 cells
ANOVA: analysis of variance
BaP: benzo[a]pyrene
COPD: chronic obstructive pulmonary disease
DAPI: 4,6-diamidino-2-phenylindole
DHE: dihydroethidium
DNMT3A: DNA methyltransferase 3A
FACS: fluorescence-activated cell sorting
FITC: fluorescein isothiocyanate
H&E: hematoxylin and eosin
HIRA-NPS: Korean Health Insurance Review and Assessment Service-National Patient Sample
IF: immunofluorescence
IGF: insulin-like growth factor
IGF2: insulin-like growth factor 2
IGF-1R: IGF1 receptor
IGF-2R: IGF2 receptor
IGFBP: IGF-binding protein
IR: insulin receptor
MLI: mean linear intercept
MMP: matrix metalloproteinase
NNK: 4-(methylnitrosamino)-1-(3-pyridyl)-1-butanol
PMN: polymorphonuclear neutrophil
RBC: red blood cell
ROS: reactive oxygen species
SC: stem cell
SPF: specific pathogen-free
TC: tobacco carcinogen
TS: tobacco smoking
TSE: tobacco smoking extract
TUNEL: terminal deoxynucleotidyl transferase dUTP nick end labeling
VDCC: voltage-dependent calcium channel

## References

[1] Collaborators GBDCRD (2020). Prevalence and attributable health burden of chronic respiratory diseases, 1990–2017: a systematic analysis for the Global Burden of Disease Study 2017. Lancet Respir Med..

[2] Celli BR, Wedzicha JA (2019). Update on clinical aspects of chronic obstructive pulmonary disease. N Engl J Med..

[3] Barnes PJ, Burney PG, Silverman EK, Celli BR, Vestbo J, Wedzicha JA (2015). Chronic obstructive pulmonary disease. Nat Rev Dis Primers..

[4] Li Y, Swensen SJ, Karabekmez LG, Marks RS, Stoddard SM, Jiang R (2011). Effect of emphysema on lung cancer risk in smokers: a computed tomography-based assessment. Cancer Prev Res (Phila)..

[5] Aamli Gagnat A, Gjerdevik M, Gallefoss F, Coxson HO, Gulsvik A, Bakke P (2017). Incidence of non-pulmonary cancer and lung cancer by amount of emphysema and airway wall thickness: a community-based cohort. Eur Respir J..

[6] Papi A, Casoni G, Caramori G, Guzzinati I, Boschetto P, Ravenna F (2004). COPD increases the risk of squamous histological subtype in smokers who develop non-small cell lung carcinoma. Thorax..

[7] Houghton AM (2013). Mechanistic links between COPD and lung cancer. Nat Rev Cancer..

[8] Ravegnini G, Sammarini G, Hrelia P, Angelini S (2015). Key genetic and epigenetic mechanisms in chemical carcinogenesis. Toxicol Sci..

[9] Schaal C, Chellappan SP (2014). Nicotine-mediated cell proliferation and tumor progression in smoking-related cancers. Mol Cancer Res..

[10] Hogan BL, Barkauskas CE, Chapman HA, Epstein JA, Jain R, Hsia CC (2014). Repair and regeneration of the respiratory system: complexity, plasticity, and mechanisms of lung stem cell function. Cell Stem Cell..

[11] Parekh KR, Nawroth J, Pai A, Busch SM, Senger CN, Ryan AL (2020). Stem cells and lung regeneration. Am J Physiol Cell Physiol..

[12] Desai TJ, Brownfield DG, Krasnow MA (2014). Alveolar progenitor and stem cells in lung development, renewal and cancer. Nature..

[13] Butler JP, Loring SH, Patz S, Tsuda A, Yablonskiy DA, Mentzer SJ (2012). Evidence for adult lung growth in humans. N Engl J Med..

[14] Kumar PA, Hu Y, Yamamoto Y, Hoe NB, Wei TS, Mu D (2011). Distal airway stem cells yield alveoli in vitro and during lung regeneration following H1N1 influenza infection. Cell..

[15] Pollak MN, Schernhammer ES, Hankinson SE (2004). Insulin-like growth factors and neoplasia. Nat Rev Cancer..

[16] Yang K, Wang X, Zhang H, Wang Z, Nan G, Li Y (2016). The evolving roles of canonical WNT signaling in stem cells and tumorigenesis: implications in targeted cancer therapies. Lab Invest..

[17] Nusse R (2008). Wnt signaling and stem cell control. Cell Res..

[18] Ziegler AN, Schneider JS, Qin M, Tyler WA, Pintar JE, Fraidenraich D (2012). IGF-II promotes stemness of neural restricted precursors. Stem Cells..

[19] Boo HJ, Min HY, Park CS, Park JS, Jeong JY, Lee SY (2023). Dual impact of IGF2 on alveolar stem cell function during tobacco-induced injury repair and development of pulmonary emphysema and cancer. Cancer Res..

[20] Gatta D, Aliprandi G, Pini L, Zanardini A, Fredi M, Tantucci C (2011). Dynamic pulmonary hyperinflation and low grade systemic inflammation in stable COPD patients. Eur Rev Med Pharmacol Sci..

[21] Oudijk EJ, Lammers JW, Koenderman L (2003). Systemic inflammation in chronic obstructive pulmonary disease. Eur Respir J Suppl..

[22] Hogg JC (2004). Pathophysiology of airflow limitation in chronic obstructive pulmonary disease. Lancet..

[23] Kwak HG, Lim HB (2014). Inhibitory effects of Cnidium monnieri fruit extract on pulmonary inflammation in mice induced by cigarette smoke condensate and lipopolysaccharide. Chin J Nat Med..

[24] Mitzner W (2008). Use of mean airspace chord length to assess emphysema. J Appl Physiol (1985).

[25] Boo HJ, Min HY, Jang HJ, Yun HJ, Smith JK, Jin Q (2016). The tobacco-specific carcinogen-operated calcium channel promotes lung tumorigenesis via IGF2 exocytosis in lung epithelial cells. Nat Commun..

[26] Livak KJ, Schmittgen TD (2001). Analysis of relative gene expression data using real-time quantitative PCR and the 2(T)(-Delta Delta C) method. Methods..

[27] Hecht SS, Isaacs S, Trushin N (1994). Lung tumor induction in A/J mice by the tobacco smoke carcinogens 4-(methylnitrosamino)-1-(3-pyridyl)-1-butanone and benzo[a]pyrene: a potentially useful model for evaluation of chemopreventive agents. Carcinogenesis..

[28] Barnes PJ (2020). Oxidative stress-based therapeutics in COPD. Redox Biol..

[29] Sun X, Kaufman PD (2018). Ki-67: more than a proliferation marker. Chromosoma..

[30] Kulkarni RN, Bruning JC, Winnay JN, Postic C, Magnuson MA, Kahn CR (1999). Tissue-specific knockout of the insulin receptor in pancreatic beta cells creates an insulin secretory defect similar to that in type 2 diabetes. Cell..

[31] Durham AL, Adcock IM (2015). The relationship between COPD and lung cancer. Lung Cancer..

[32] Young RP, Hopkins RJ (2010). Link between COPD and lung cancer. Respir Med..

[33] Barnes PJ, Adcock IM (2011). Chronic obstructive pulmonary disease and lung cancer: a lethal association. Am J Respir Crit Care Med..

[34] Franco R, Schoneveld O, Georgakilas AG, Panayiotidis MI (2008). Oxidative stress, DNA methylation and carcinogenesis. Cancer Lett..

[35] Cushing L, Jiang Z, Kuang P, Lu J (2015). The roles of microRNAs and protein components of the microRNA pathway in lung development and diseases. Am J Respir Cell Mol Biol..

[36] Watanabe N, Fujita Y, Nakayama J, Mori Y, Kadota T, Hayashi Y (2022). Anomalous epithelial variations and ectopic inflammatory response in chronic obstructive pulmonary disease. Am J Respir Cell Mol Biol..

[37] Chen Q, Hirai H, Chan M, Zhang J, Cho M, Randell SH (2024). Characterization of perivascular alveolar epithelial stem cells and their niche in lung homeostasis and cancer. Stem Cell Rep.

[38] Wang C, Hyams B, Allen NC, Cautivo K, Monahan K, Zhou M (2023). Dysregulated lung stroma drives emphysema exacerbation by potentiating resident lymphocytes to suppress an epithelial stem cell reservoir. Immunity..

[39] Katsura H, Sontake V, Tata A, Kobayashi Y, Edwards CE, Heaton BE (2020). Human lung stem cell-based alveolospheres provide insights into SARS-CoV-2-mediated interferon responses and pneumocyte dysfunction. Cell Stem Cell..

[40] Blyth AJ, Kirk NS, Forbes BE (2020). Understanding IGF-II action through insights into receptor binding and activation. Cells..

[41] LaCanna R, Liccardo D, Zhang P, Tragesser L, Wang Y, Cao T (2019). Yap/Taz regulate alveolar regeneration and resolution of lung inflammation. J Clin Invest..

[42] Kim WY, Jin Q, Oh SH, Kim ES, Yang YJ, Lee DH (2009). Elevated epithelial insulin-like growth factor expression is a risk factor for lung cancer development. Cancer Res..

[43] Chubachi S, Takahashi S, Tsutsumi A, Kameyama N, Sasaki M, Naoki K (2017). Radiologic features of precancerous areas of the lungs in chronic obstructive pulmonary disease. Int J Chron Obstruct Pulmon Dis..

[44] Laniado-Laborin R (2009). Smoking and chronic obstructive pulmonary disease (COPD). Parallel epidemics of the 21 century. Int J Environ Res Public Health..

[45] Parris BA, O'Farrell HE, Fong KM, Yang IA (2019). Chronic obstructive pulmonary disease (COPD) and lung cancer: common pathways for pathogenesis. J Thorac Dis..

[46] Elliott WJ, Ram CV (2011). Calcium channel blockers. J Clin Hypertens (Greenwich)..

