# Supplementary figures and images for: Autocrine insulin-like growth factor 2 signaling as a potential target in the associated development of pulmonary emphysema and cancer in smokers
Source: Inflamm Regen. 2024 Jun 21;44:31. doi: 10.1186/s41232-024-00344-3 (PMC11191215; doi:10.1186/s41232-024-00344-3)

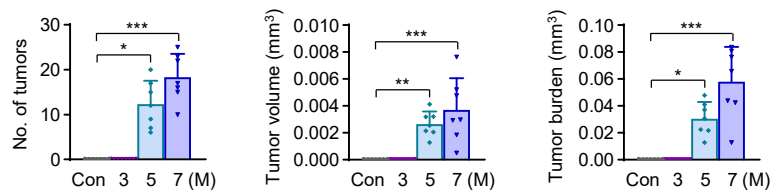

Supplement: Supplementary file 1 — Additional file 1: Supplementary Fig. 1. Quantitative analyses of tumor formation NB-treated mice (n = 7/group). Data are the mean ± SD. *p < 0.05; **p < 0.01; ***p < 0.001 (Kruskal-Wallis test with Dunn’s post-hoc test). [file 41232_2024_344_MOESM1_ESM.pdf]

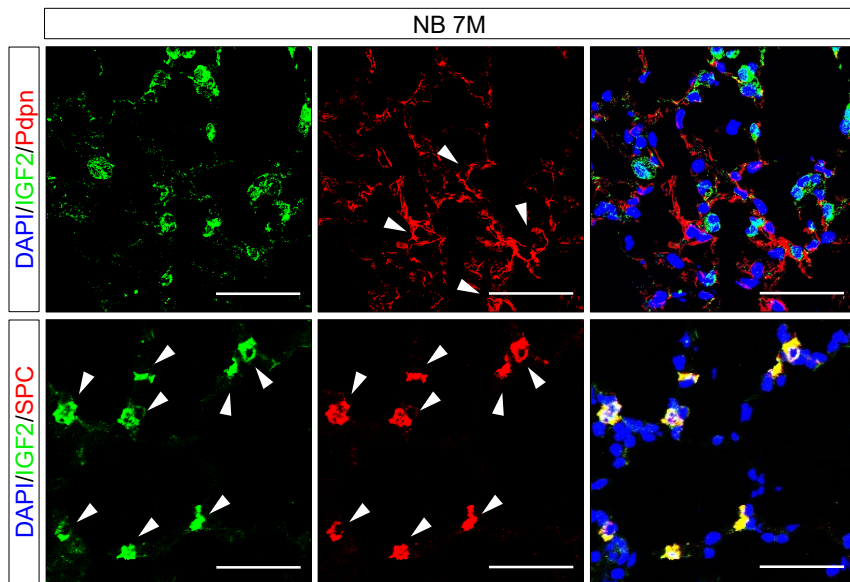

Supplement: Supplementary file 2 — Additional file 2: Supplementary Fig. 2. Representative IF images showing IGF2 expression in SPC+ AT2s and Pdpn+ AT1s. Scale bars: 50 μm. [file 41232_2024_344_MOESM2_ESM.pdf]

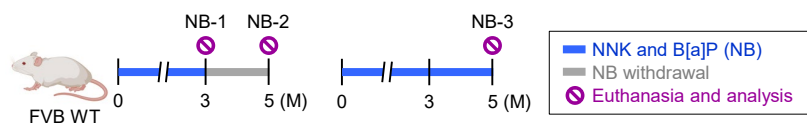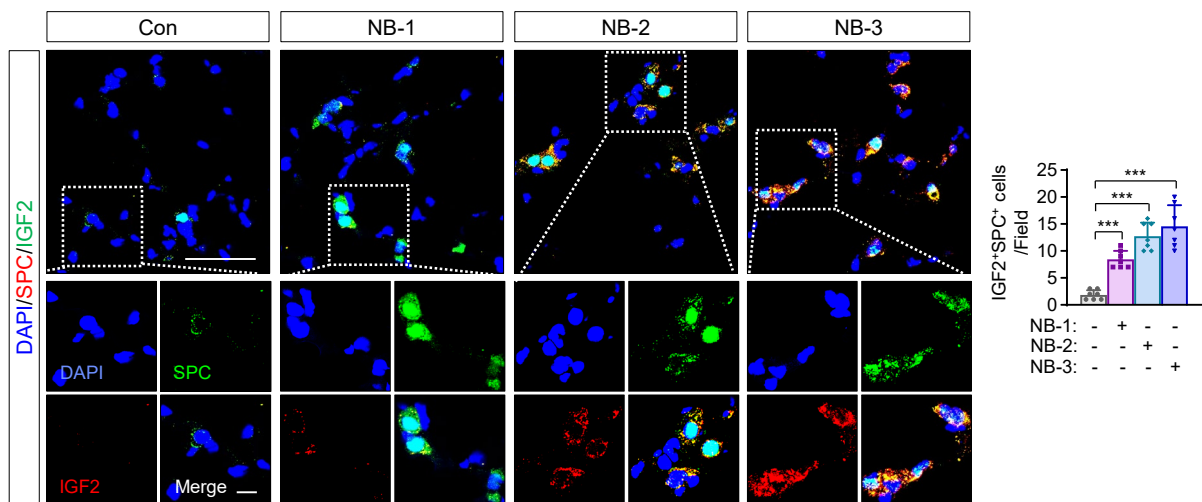

Supplement: Supplementary file 3 — Additional file 3: Supplementary Fig. 3. Representative IF images and quantitative analysis (n = 7) showing the regulation of IGF2 expression in mice treated with NB for 3 months, those treated with NB for 3 months and withdrawn from NB treatment for 2 months, or those treated with NB for 5 months. Data are the mean ± SD. *p < 0.05; **p < 0.01; ***p < 0.001 (one-way ANOVA with Dunnett’s post-hoc test). Scale bars: 50 μm. 10 μm (insets). [file 41232_2024_344_MOESM3_ESM.pdf]
