# Supplementary material for: Autocrine insulin-like growth factor 2 signaling as a potential target in the associated development of pulmonary emphysema and cancer in smokers
Source: Inflamm Regen. 2024 Jun 21;44:31. doi: 10.1186/s41232-024-00344-3 (PMC11191215; doi:10.1186/s41232-024-00344-3)
Supplement: Supplementary file 4 — Additional file 4: Supplementary Table 1. Demographic characteristics of the study population. [file 41232_2024_344_MOESM4_ESM.pdf]

Supplementary Table 1. Demographic characteristics of the study population*

Characteristics	Number of patients**							
	NPS 2012		NPS 2013		NPS 2014		NPS 2012-2014	
	Unweighted	Weighted	Unweighted	Weighted	Unweighted	Weighted	Unweighted	**Weighted**
Total patients	1,121,751	37,391,232	1,138,381	37,945,622	1,152,073	38,402,013	3,412,205	**113,738,867**
Gender								
**Male**	534,694	17,822,884	543,894	18,129,563	550,898	18,363,039	1,629,486	**54,315,486**
**Female**	587,057	19,568,347	594,487	19,816,059	601,175	20,038,975	1,782,719	**59,423,381**
Age (years old)								
**19 - 34**	307,335	10,244,412	307,304	10,243,353	306,192	10,206,314	920,831	**30,694,079**
**35 – 49**	348,226	11,607,437	347,159	11,571,905	346,031	11,534,242	1,041,416	**34,713,584**
**50 – 64**	288,176	9,605,729	297,731	9,924,224	305,700	10,189,896	891,607	**29,719,849**
**65 - 79**	144,552	4,818,288	150,020	5,000,587	154,903	5,163,350	449,475	**14,982,225**
**80 and older**	33,462	1,115,366	36,167	1,205,551	39,247	1,308,212	108,876	**3,629,129**
Public insurance								

scheme								
**Health insurance**	1,079,950	35,997,888	1,097,753	36,591,373	1,112,269	37,075,230	3,289,972	**109,664,491**
**Medicaid**	38,539	1,284,613	37,214	1,240,451	36,544	1,218,118	112,297	**3,743,182**
**Veteran healthcare**	3,262	108,731	3,414	113,798	3,260	108,665	9,936	**331,194**
Calcium channel blocker (CCB) used								
**No**	1,011,249	33,707,901	1,031,246	34,374,505	1,051,520	35,050,291	3,094,015	**103,132,697**
**Dihydropyridine**	101,518	3,383,870	98,046	3,268,155	91,265	3,042,126	290,829	**9,694,151**
**Non-dihydropyridine**	8,984	299,461	9,089	302,962	9,288	309,596	27,361	**912,019**
COPD	13,653	455,090	12,926	430,860	12,698	423,260	39,277	**1,309,210**
Emphysema	2,450	80,165	2,449	81,632	2,512	83,732	7,366	**245,529**
Pneumothorax	1,051	35,033	1,061	35,366	990	33,000	3,102	**103,398**
Hypertension	246,246	8,208,050	254,652	8,488,285	261,774	8,725,687	762,672	**25,422,023**
Angina Pectoris	57,437	1,914,531	60,427	2,014,205	64,287	2,142,871	182,151	**6,071,607**

*study population is the patient who is aged 19 or over

**in millions
